# Supplementary material for: Retinotopic biases in contextual feedback signals to V1 for object and scene processing
Source: Curr Res Neurobiol. 2024 Nov 23;8:100143. doi: 10.1016/j.crneur.2024.100143 (PMC11731975; doi:10.1016/j.crneur.2024.100143)
Supplement: Multimedia component 1 [file mmc1.pdf]

Peer Review Overview

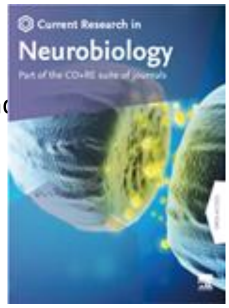

Manuscript Title: Retinotopic biases in contextual feedback signals to V1 for object and scene processing

Received	Jan 26, 2024
1st Decision	Apr 09, 2024
1st Revision Submitted	July 18, 2024
2nd Decision	Sep 09, 2024
2nd Revision Submitted	Sep 27, 2024
Accepted	Oct 08, 2024

1st Decision letter

Reference: CRNEUR-D-24-00001

Title: Retinotopic biases in contextual feedback signals to V1 for object and scene processing

Journal: Current Research in Neurobiology

Dear Prof Muckli,

Thank you for submitting your manuscript to Current Research in Neurobiology.

The reviewers have raised concerns that would need to be considered and I recommend reconsideration of your manuscript following a major revision. I invite you to resubmit your manuscript after addressing the comments below. Please resubmit your revised manuscript by **Jun 08, 2024**.

When revising your manuscript, please consider all issues mentioned in the reviewers' comments carefully: please outline every change made in response to their comments and provide suitable rebuttals for any comments not addressed. Please note that your revised submission may need to be re-reviewed.

Current Research in Neurobiology values your contribution and I look forward to receiving your revised manuscript.

Kind regards,

Christopher I. Petkov
Editor in Chief
Current Research in Neurobiology

Comments from Editors and Reviewers:

Reviewer #1:

The manuscript presents a first fMRI study, where Ps are shown peripherally presented objects embedded in scenes and are required to indicate (1) whether the objects are the same or (2) the nature of the scene (when a particular boundary is presented). They occlude peripheral and central parts of the display. They flash checkboards in these occluded central and peripheral portions to generate ROIs corresponding to these locations in V1 and then used linear SVMs to decode the two scene and two object types on the basis of that central or peripheral V1 information.

When Ps performed the scene task, the scene could be decoded in peripheral and foveal occluded V1 regions. When performing the object task, it could not. This suggests that scene information is fed back to foveal and peripheral V1 regions only when performing the scene task. Objects could not be decoded in either location in either task. In fact though, objects could not be decoded well from directly stimulated regions of V1 either - only just above chance in the object task and not decodable in the scene task.

The authors reasoned that the unnatural nature of objects, and difficult nature of the task, may have disrupted the object decoding. Exp 2 was therefore designed with more natural object-scene pairs, such that they were always congruent with the scene - BBQ/tent on a beach/forest. Ps now performed an object discrimination task (tent, BBQ, nothing) or scene discrimination (beach, forest, nothing). Scenes could be decoded regardless of task in foveal and peripheral occluded regions, and objects in foveal V1 only during the object task (and neither task in peripheral V1).

The broad question here is fascinating. Within predictive coding schemes, it is assumed that information is passed reciprocally up/ down the hierarchy to convey predictions and errors with respect to those predictions, but we know little about the information communicated. These occlusion paradigms are a useful tool for shedding some light on this information. The idea that there may be some distinctions in foveal vs peripheral processing for different types of information is also fascinating. I therefore recommend publication but just have a few questions that would help clarify these findings and their significance for readers:

More important points:

The authors discuss how the higher resolution of the fovea may be important for distinguishing object features, and therefore we may typically act to bring these to the fovea via saccades. Hence perhaps the fovea is important for object discrimination because saccadic remapping generates V1 foveal effects even though the object is never brought to the fovea in this task. This is an interesting idea. I was left wondering on two dimensions though. First, foveal effects are also found for scenes, so why does the discussion focus on object vs scene differences? Second, would such a mechanism really distinguish objects vs scenes per se, or instead the precision necessary for the task? E.g., one might assume that forest vs beach can be distinguished via textural information that can be determined peripherally. Tents vs BBQs perhaps less so. But there is other contextual/scene information that would benefit from the resolution at the fovea. E.g. if asked whether the street is in LA or Rothenburg, perhaps I need to foveate the surface to determine whether it's cobble or tarmac. And distinguishing bananas and apples I can probably do in my periphery. So would mechanistic differences reflect the necessary precision for identification rather than anything intrinsic to visual category?

The authors argue that this scene processing is disrupted by a difficult object discrimination task, but I

wonder whether they could consider other possibilities here? Could it also be that scenes must be task relevant to decode scenes?

The authors also conclude that objects can *only* be decoded from foveal V1 if task relevant, but I feel this conclusion should be toned down. The fact this effect is *stronger* when task relevant seems perhaps more likely to replicate. The reason for my reservation in claiming it would only be seen at all when task relevant is twofold. First, the sample sizes here are small (10 in Exp 1 and 18 in Exp 2) and therefore I feel nervous about drawing too much from absent effects. Perhaps just underpowered. Second, decoding of objects in Exp 1 is low also for directly stimulated regions, likely because these are unusual objects.

The processes are described as active, partly due to task relevance differences. But does task relevance increase decoding from stimulated regions too? I.e. objects decoded from stimulated V1 regions with higher accuracy when performing the object than scene task? In which case, may the feedback to V1 be determined by level of information fed forward rather than the task relevance itself? If this analysis is easy to perform/add it would be informative with respect to this issue, but if not, perhaps this difference in feedforward processing could also be considered in the discussion.

Smaller points:
1. How were the sample sizes determined? 10 in Exp 1 seems rather low. Did they terminate early because it was clear that the object decoding was unsatisfactory?
2. The stimuli flash at a rate of 4 Hz. Why was this? What was presented in the interstimulus intervals and how long were they?
3. Figs 2 and 4 give classification accuracy as values minus 50%. But the mean values in each condition are ~0.03. Given the text I infer that these values are proportions rather than percentages, but this is confusing when labelled as "-50%". Should all labels be converted to %ages?
4. I struggled to make out Fig 1A. Could it be made larger so we can get an idea of the particular scene and objects presented?

Reviewer #2:

Bennett and colleagues present an extension of earlier work from the same that has demonstrated decoding of information in parts of V1 that correspond to an occluded portion of a scene. Those results have been interpreted as showing feedback from higher level cortex and here the authors ask how feedback varies according to stimulus type (object, scene), task (object, scene) and eccentricity in V1 (fovea, periphery). They report that scene information is fed back to both foveal and peripheral cortex, but can be disrupted by an object task. In contrast, they find that object information is only fed back to foveal cortex and only with an object task.

While the study addresses an interesting question, the results are not particularly compelling. The results of the experiments are analyzed only with limited specific comparisons, the effect sizes seem quite small, the stimulus conditions tested are very limited, and the two experiments are quite different in the nature of the stimuli and task making it challenging to draw any reliable conclusions from their comparison. Overall, this means that my confidence in the overall interpretation and conclusions is very low.

Specific Comments -

Experiment 1: the design of Experiment 1 is essentially a 2 (ROIs) x 2 (information type) x 2 (task) design, yet the authors only report selected paired comparisons. The Bonferroni correction that was applied only considered 8 comparisons (presumably each condition versus chance) but the authors also report comparisons between conditions. It strikes me that it would be more appropriate to conduct something like an ANOVA or equivalent, considering the nature of the design.

Experiment 2: again, it seems strange to analyze the factorial design with just a select few tests. The Bonferroni correction that was applied considers 16 tests, which means that no comparisons between conditions are considered even though such comparisons are mentioned in the text.

Limited stimuli: In Experiment 1 there were just two scene images and two (arbitrary) object types. In Experiment 2, there were just two scene images and two object images (at least the text does not indicate the use of multiple scene/object exemplars). Further, it seems that the occluded portion of the visual field was always the same. Such limited stimuli raise the concern that the results may not be generalizable and may instead reflect the specific nature of the stimuli. In part, Experiment 2 was run to address such concerns about the arbitrary object stimuli, but focusing on BBQs and tents (particularly if there was only one exemplar of each object) may suffer from the same problem. The results would be more compelling if a broader range of stimuli had been tested.

Feedback: since the experiment is testing feedback into V1, it would be useful to show that object information is present in higher areas, especially since object decoding was very weak in Experiment 1 even in the feedforward stimulated portion of V1. Can the object be decoded in, for example, LOC? Even more useful would be to present searchlight analyses for both experiments, showing where object information can be decoded across visual cortex.

Methods: "a FOV of 220mm, flip angle of 62° and a resolution of 3.0mm3". From this description it's ambiguous what the voxel size was. Was the volume of each voxel 3.0mm3? I don't think that's what the authors mean. Providing the matrix size would make this clearer.

ROIs: Given the nature of the foveal confluence, can the authors really state that they have identified foveal V1. Would it be more accurate to state that it is 'foveal early visual cortex' and might include V2 and V3.

Figure 2: the y-axis label and the numbered tick marks don't match - presumably they should both be in proportions or both in percentages.

Experiment 1 - object stimuli: it's currently very hard to see the nature of the object stimuli in Experiment 1. Larger images either in the main manuscript or in supplementary material would be very helpful.

Reviewer #3:

This manuscript describes two fMRI experiments aimed at testing foveal fMRI responses to peripherally-presented stimuli of objects and scenes. The goal of the study was to understand the role of feedback from higher-level visual cortex to the fovea of early visual cortex. While this study is an interesting follow-up to a landmark (and, as yet, unreplicated) experiment from the Kanwisher lab, the authors have presented a very cursory analysis of the data that unfortunately makes it impossible to draw any

firm conclusions. While it is conceivable that a deeper dive into the data would permit solid conclusions and render this a more compelling study, it is simply not possible to evaluate whether there is anything of interest here given the results as currently presented.

Below I highlight some of the more salient issues that the authors may want to consider in developing this study further.

1. Figure 1 illustrates the approach the authors used to select voxels with "pRFs falling entirely within the occluded region." The logic behind this analysis is that voxels with pRF inside the occluded region do not respond to the object or scene stimuli as a result of feedforward drive. Hence, activity in these voxels should instead be attributed to feedback. While I realize that this logic has been followed by many previous studies from this lab, this logic is problematic for the following two reasons.

First, pRF's are statistical estimates of a voxel's spatial profile. A pRF's size is a useful metric only to the extent that the model describes the true underlying hemodynamic response. pRF's are typically estimated with a two-dimensional Gaussians. But is a Gaussian the best model for describing visual pRFs in visual cortex? There is growing evidence that it is not. For example, pRFs may have an elliptical shape, elongated and pointing toward the fovea. pRFs have also been described as having spatially-extensive suppressive surrounds. Finally, pRFs may also have heavy tails, with a spatial extent that far exceeds that described by the FWHM of a 2D Gaussian. Any of these alternative pRF models could result in the inclusion of voxels that directly responded to the scene and object stimuli. Moreover, the FWHM of a 2D Gaussian is an arbitrary cutoff to describe the pRF size. Obviously, a voxel can respond meaningfully to stimuli presented more than 2 sigma from their centers, and MVPA can be sensitive to such responses, however small, when aggregated over many voxels.

Second, including voxels in the analysis that do not respond to the checkboard stimulus commits the statistical fallacy of accepting the null hypothesis (that there is no response). In other words, while it is statistically valid to use a localizer to include voxels that significantly respond to a stimulus, it is not valid to include voxels because they did not significantly respond to the stimulus (as the checkerboard localizer essentially does). It would be far better to simply plot object selectivity as a function of pRF location.

In essence, I am concerned that what the authors would like to attribute to feedback may simply be due to feedforward stimulus drive. Of course, this critique also applies to the original Williams paper and has not been addressed adequately.

2. Figure 2 shows a failure to replicate the classic Williams et al. effect of classifying object identity at the fovea. It also fails to replicate the Williams finding of classifying at the portion of V1 corresponding to the retinotopic location of the stimuli. The authors have a reasonable, if not entirely convincing, explanation for this discrepancy. However, given these two negative results, one would really like to see a convincing positive result to ensure that they authors are doing the experiment properly (as a million things can go wrong, from task design, data acquisition, response estimation, through MVPA). The authors should be able to demonstrate basically ceiling-level decoding in object- and scene-selective cortex on the ventral and lateral surface of the brain for object and scene stimuli.

A few small comments on Fig 2. The y-axis of Fig 2 is adjusted by subtracting 50% (chance) decoding, but the units are expressed in proportion correct, which is confusing. The authors should use permutation tests to estimate a null distribution (rather than depend on parametric tests). Its impossible to tell

whether any of this is in fact significant. Finally, it's perplexing that performance of the scene task boosts decoding for objects. This had me wondering about eye movements (where subjects making more saccades in the scene task, generally boosting decoding of everything?).

3. Given that the results from Expt 1 were generally not significant, the authors conducted Expt 2, with different object categories and scene stimuli, crossed again with object or scene tasks. The results are perplexing. In Expt 2, the authors could decode scene from both ROIs, in both tasks (in contrast to Expt 1). One wonders where in the brain it is *not* possible to decode the scene stimuli in this experiment? More problematic is the over reliance on very small differences in decoding accuracy to support big claims about where in the brain information is processed. I am simply not comfortable with saying that "Tent during Object Task: 51.9%, p<0.001" is significant, but that "Tent during Scene Task: 51.5%, p=0.0678" is not, when they basically have the same mean, but fall just north or south of an arbitrary p-value threshold that probably depends greatly on arbitrary analysis decisions (e.g., which classifier was used), and that does not meet the basic distributional assumptions of the parametric test employed.

Reviewer #4:

GENERAL COMMENTS

In this study, the authors used fMRI to image cortical responses to visual stimuli presenting occluded regions in the foveal or peripheral parts of the visual field, while subjects performed scene or object recognition tasks. Using multivoxel pattern classification, they could decode object information in the foveal region only, but scene information from both fovea and periphery. From this result they draw some complicated conclusions about how detailed object information vs automatic background information are processed in the brain. I find that the study is somewhat weak in the stimulus design and lack of adequate controls to interpret the results and I worry that the authors are reading too much into their results. There is also lack of detailed methodological information as explained in the comments below.

MAJOR SPECIFIC COMMENTS

1. The introductory paragraph presents the theory of predictive coding as a fact, as if it were well established that the brain performs predictive coding. In fact, this is only a theory and this should be presented as such.

2. Fig. 1A. It would be helpful to include in this figure information on the size of the image, occluded regions and the size of the object. This is important for understanding the retinotopy or lack thereof of feedback (FB) signals.

3. Results p. 3 bottom paragraph, the authors state" "We were able to decode scene information during the scene task only, in both foveal (Scene Task: 54.6%, p<0.01; Object Task: 49.6%, p=0.658)....". I do not understand these percentages. What are you comparing here? classification accuracy in the fovea for scenes during scene task and during object task? If so, please state this clearly. Moreover, these percentages do not correspond to those shown in Fig. 2. Also, the units on the y axis of Fig. 2 are very confusing. Does 0 here corresponds to 50%? Maybe say so in the legend and also the units on the y axis are in decimals while the text and the Y axis label reports percentages. Please be consistent.

4. Results p. 3 and Fig. 2 (Experiment 1). Based on the known retinotopic organization of FB connections to V1 and the specific stimulus geometry used for this experiment, scene information is fed back to both occluded regions (fovea and periphery) while object-related feedback info is relayed more to the fovea than to the periphery. Thus, when attention is directed to the object, during the object task, the FB signals arising from the region of cortex representing the object are relatively more enhanced and dominate FB signals. Due to the object size being small or "unique" (as the authors state) perhaps this is insufficient to provide robust FB signals carrying object information (insufficient for object classification) but sufficient to disrupt the FB signals coming from the scene. Is this a possible interpretation of the results of Exp. 1?

5. Results Experiment 2. Fig. 4. The authors performed control experiments (no scene or object in the stimulus) but did not report the results from these experiments in Fig. 4. These should be reported.

6. Results p. 6, 1st paragr., the authors state:" We found that object presence in the scene was detectable in foveal V1 for the Tent in the object task only". Was this only for the Tent but not the Barbecue? Why? What do the red bars in Fig. 4 represent? Object presence? while purple represents object identity? Please specify in the legend. Also please use different color bars for tent vs barbecue.

7. Results p. 2, 2nd paragr. The authors state: "Object identity information was detectable in foveal V1 only during the object task.." In reality, I do not see any difference in Fig. 4 between the bbq during object or scene tasks in either peripheral or foveal V1, so this statement is true only for the tent, but even for the tent the differences appear extremely small and perhaps are not significant.

8. It seems to me that the scene and object stimuli used in the study are not comparable. The peripheral mask is an occlusion of the scene and the FB signals can be interpreted as carrying information about the occluded region (a prediction contextual signal), but the Foveal mask is also occluding only the scene but not the object. As a result, the object task is very different from the scene task, the first one is a prediction of what is behind the occluder, the second is object recognition performed in the parafoveal region. In the object task, attention is directed to a specific parafoveal region, while in the scene condition attention is spread out all around peripheral visual field. It is difficult to know whether the differences in the results that the authors found have to do specifically with object stimuli vs scenes as opposed to the differences in the spatial allocation of attention, or differences in the kinds of tasks the subject is performing (occlusion vs recognition).

9. I cannot find any details in the methods about whether they controlled for eye movements.

10. Discussion p. 8. The last sentence of the middle section, states :" It does not help to account for the task related feedback to foveal regions in our results". I do not agree with this statement. The object, particularly in experiment 2, is not in the periphery but it occupies a wide region of visual field consistent with the feedback activated by it being capable of activating both the fovea and the periphery, just like the scene stimuli.

One possibility is that object-related FB arises from cortical areas having more precise topography and smaller receptive fields (RFs) such as V4, which also emphasize fovea, whereas scenes recruit FB from higher areas with larger RFs that emphasize the periphery. This explanation is hinted at in this section of the Discussion, but not presented explicitly as a possibility, which would not rule out that FB acts retinotopically

1st Author Response Letter

Response to comments from Editors and Reviewers:

Dear Prof Petkov,

Thank you again to you and the reviewers, and we are glad to receive your positive response. We're happy to attend to the remaining comments, providing below a description of our edits.

Best regards,

Lars Muckli

Reviewer 1: The authors have satisfactorily addressed my concerns, both via answering some conceptual questions in the text and performing some additional analyses concerning feedforward processing. I have no further comments and recommend the manuscript for publication.

Thank you.

Reviewer 2: The authors have been quite responsive in their reply and in refence to my original concerns, they have included additional analyses, specifically a linear mixed effects model analysis and a searchlight analysis. These are important additions, but they do they do not allay my concerns over the interpretation. As shown by the LME analysis, the most robust results are the stronger decoding for scenes compared to objects, and the stronger decoding in Experiment 2 compared with Experiment 1. The effects relating to eccentricity are qualitatively present but are very weak (as acknowledged by the authors). I don't think there's much the authors can do with the current data set to change this, so my suggestion is that the authors tone down their claims and their speculations, particularly in the abstract. This is an interesting study, but the authors need to acknowledge more clearly in the manuscript that the results are merely suggestive and not conclusive., making clear in the discussion what the limitations of the evidence are.

We are glad that the reviewer supports our previous revision, and we thank the reviewer for these last comments. To address the reviewer, we have toned down our claims in the abstract and discussion as suggested. Specifically, in the abstract we have removed the general claim that feedback of object information arrives to the fovea and put more emphasis on our scene decoding before object decoding. In the discussion, we again tone down the claim that object presence and/or identity is fed back to the fovea, specifically mentioning the conditions during which our results are more robust in our second experiment.

2nd Decision letter

Reference: CRNEUR-D-24-00001

Title: Retinotopic biases in contextual feedback signals to V1 for object and scene processing

Journal: Current Research in Neurobiology

Dear Prof Muckli,

I am pleased to inform you that we can provisionally accept your paper following final minor correction that can be editorially evaluated.

The reviewers recommend reconsideration of your manuscript following by one of the reviewers a further minor revision and modification as outlined below. I invite you to resubmit your manuscript after addressing the comments below. Please resubmit your revised manuscript by **Oct 09, 2024**.

When revising your manuscript, please consider all issues mentioned in the reviewers' comments carefully: please outline every change made in response to their comments and provide suitable rebuttals for any comments not addressed. Please note that your revised submission may need to be re-reviewed.

Current Research in Neurobiology values your contribution and I look forward to receiving your revised manuscript.

Kind regards,

Christopher I. Petkov
Editor in Chief
Current Research in Neurobiology

Comments from Editors and Reviewers:

Reviewer #1:

The authors have satisfactorily addressed my concerns, both via answering some conceptual questions in the text and performing some additional analyses concerning feedforward processing. I have no further comments and recommend the manuscript for publication.

Reviewer #2:

The authors have been quite responsive in their reply and in refence to my original concerns, they have included additional analyses, specifically a linear mixed effects model analysis and a searchlight analysis. These are important additions, but they do they do not allay my concerns over the interpretation. As shown by the LME analysis, the most robust results are the stronger decoding for scenes compared to objects, and the stronger decoding in Experiment 2 compared with Experiment 1. The effects relating to

eccentricity are qualitatively present but are very weak (as acknowledged by the authors). I don't think there's much the authors can do with the current data set to change this, so my suggestion is that the authors tone down their claims and their speculations, particularly in the abstract. This is an interesting study, but the authors need to acknowledge more clearly in the manuscript that the results are merely suggestive and not conclusive., making clear in the discussion what the limitations of the evidence are.

Accept Letter

Dear Prof Muckli,

Thank you for submitting your manuscript to Current Research in Neurobiology.

I am pleased to inform you that your manuscript has been accepted for publication.

My comments, and any reviewer comments, are below.

Your accepted manuscript will now be transferred to our production department. We will create a proof which you will be asked to check, and you will also be asked to complete a number of online forms required for publication. If we need additional information from you during the production process, we will contact you directly.

We appreciate and value your contribution to Current Research in Neurobiology. We regularly invite authors of recently published manuscript to participate in the peer review process. If you were not already part of the journal’s reviewer pool, you have now been added to it. We look forward to your continued participation in our journal, and we hope you will consider us again for future submissions.

CRNEUR aims to be a unique, community-led journal, as highlighted in the Editorial Introduction. As part of this vision, we will be regularly seeking input from the scientific community and encourage you and your co-authors to take the survey.

We would also like to invite you to take part in our CRNEUR Author Question & Answer (Q&A), which could get published alongside your article and help to promote it. We suspect you might have an interesting story of perseverance or team work that was required for the research study to complete, or a diversity of perspectives that you might share, as a way of inspiring others about neuroscience.

Kind regards,

Christopher I. Petkov
Editor in Chief
Current Research in Neurobiology

-------- End of Review Comments --------
